# ALK-Positive Histiocytosis: A Case Report and Literature Review

**DOI:** 10.5146/tjpath.2020.01507

**Published:** 2021-05-15

**Authors:** Omar Issa Jaber, Doa’ Al Jarrah, Mohammad Hiasat, Maysa Al Hussaini

**Affiliations:** Department of Pathology and Laboratory Medicine, King Hussein Cancer Center, Amman, Jordan; Neuron Clinics, Amman, Jordan

**Keywords:** Histiocytosis, Anaplastic lymphoma kinase (ALK), Adult, Spinal cord

## Abstract

ALK positive histiocytosis is a relatively new histiocytic proliferation disease with a characteristic gene translocation involving fusion of the *ALK* gene with different partners, mostly *KIF5B.* We report a case of ALK-positive histiocytosis with literature review. A 27-year-old male patient presented mainly with progressive lower limb weakness. Imaging studies showed an intradural extramedullary enhancing lesion at the L3 level. A 1.5 cm mass was excised from the sensory nerve root in the filum terminale at the level of L3. Histologic examination showed infiltration of the nerve by numerous histiocytes with moderate to abundant eosinophilic to clear-foamy and variably-vacuolated cytoplasm with irregular-to-smooth contoured nuclei. The histiocytes were positive for CD68 and ALK1 and negative for S100 and CD1a. *KIF5B-ALK* fusion was detected by real time-polymerase chain reaction. The patient is asymptomatic nine months after surgical excision. This is the first reported localized case occurring in the nerve root of an adult patient, thus expanding the clinical manifestations of this disease. An integrated histological, immunohistochemical and molecular approach is recommended for diagnosis. We recommend performing ALK1 immunohistochemical stain on all histiocytosis cases to increase awareness and detection of this newly described entity.

## INTRODUCTION

ALK-positive histiocytosis is a relatively new histiocytic proliferation disease with a characteristic gene translocation involving fusion of the *ALK* gene with different partners, mostly *KIF5B*. The first reported cases were infants who presented with systemic manifestations including pallor, anemia, thrombocytopenia, and hepatosplenomegaly (1). A few years later, the same group expanded their cohort by reporting seven additional cases that involved older patients (age: 2-40 years old) with systemic as well as localized disease (2). In this article, we report a case of ALK-positive histiocytosis localized to the sensory nerve root of a 27-year-old man who presented with neurologic symptoms manifesting mainly as progressive lower limb weakness. Confirmatory molecular testing detected the presence of *ALK-KIF5B* gene fusion.

## CASE REPORT

**Figure 1:**
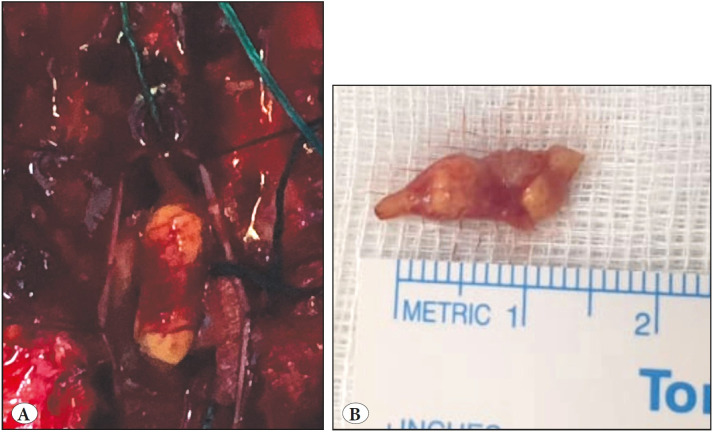
**A)** Intra-operative view of the lesion at the filum terminale. **B)** Gross appearance of the lesion after excision.

A 27-year-old male patient, not previously known to have any medical chronic disease, presented complaining of a 4-month history of progressive left lower limb weakness, low back pain, and left calf neuropathic pain. Physical examination showed left plantar flexion weakness (4/5), left extensor halluces longus (EHL) paralysis (0/5), and left knee flexion weakness (4/5). Lumbar spine MRI showed an intradural extramedullary enhancing lesion at the L3 level. Lumbar spine MRI with contrast revealed a small enhancing nodule involving the filum terminale at the level of L3, with an isodense signal in T1WI, an intermediate signal in T2WI, and bright signal post-contrast images. Radiologically, the main differential diagnosis was ependymoma. The patient underwent surgery (a two-level laminectomy of L2 and L3, and gross total resection of the intradural mass arising from the sensory nerve root after releasing it from two motor nerve roots contained within the arachnoid band). Intraoperative neuromonitoring showed improvement in the motor function of the left L5 and S1 nerve roots. The post-operative course was uneventful with no new neurological deficits. The previous neurological deficits significantly improved and he was discharged 2 days following surgery (Figure 1).

Further workup that included a CT of the neck, chest, abdomen, and pelvis in addition to bone scan did not show any abnormalities. Nine months post-operatively the patient was completely asymptomatic and regained full power in the lower limbs. Contrasted lumbar spine MRI showed no recurrence.

**Figure 2:**
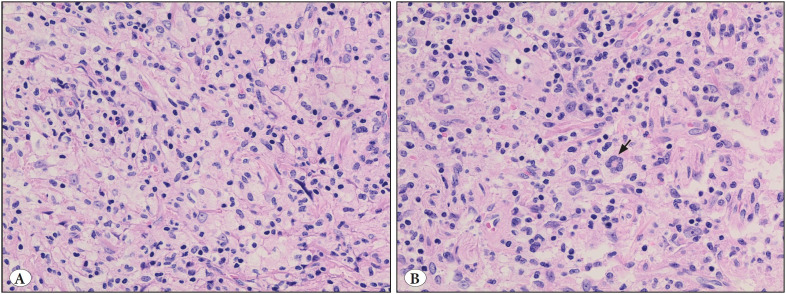
**A)** The histiocytes have eosinophilic to clear cytoplasm with round to irregular nuclei and occasional prominent nucleoli (H&E; x400). **B)** A Touton-type multinucleated giant cell (arrow) (H&E; 400).

**Figure 3:**
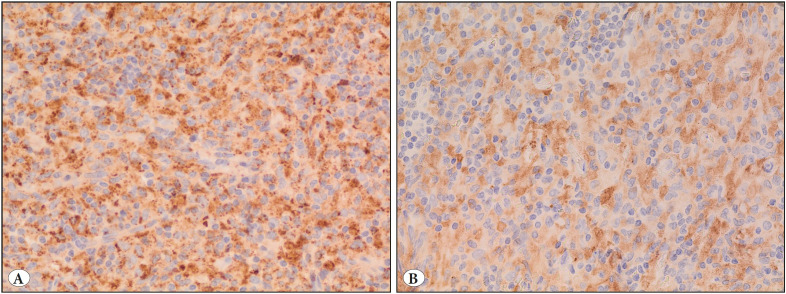
**A)** CD68 expression in ALK-positive histiocytosis (IHC; x400). **B)** ALK1 expression in ALK-positive histiocytosis (IHC; x400).

We received the case as a consultation following an outside pathology diagnosis of Langerhans cell histiocytosis (eosinophilic granuloma). Histologic examination showed nerve tissue infiltrated by histiocytes with moderate to abundant eosinophilic to clear-foamy and variably-vacuolated cytoplasm. The nuclei ranged from oval in shape to grooved with irregular outlines (Figure 2). They had fine chromatin with small nucleoli. Chronic lymphocytic cell infiltrates were seen in the background. Rare Touton-type multinucleated giant cells were found (Figure 2) and rare mitotic figures were seen (1/10 HPFs). No necrosis was present. The histiocytes were positive for CD68 (Figure 3) and negative for S100 protein, CD1a, and BRAF V600E immunostains. ALK-1 immunohistochemical stain showed diffuse positivity in the histiocytes (Figure 3). Therefore, ALK-positive histiocytosis was suspected and confirmatory molecular testing for the presence of *ALK* gene translocation, which was performed at an outside facility, showed the presence of *KIF5B-ALK* gene fusion by RT-PCR, confirming the diagnosis.

## DISCUSSION

ALK-positive histiocytosis is a relatively recent entity among the histiocytic disorders. The first series was published in 2008 and the reported three cases were neonates and infants who presented with pallor, hepatosplenomegaly, anemia, and thrombocytopenia. The organs involved included the liver, spleen, bone marrow, and skin and showed infiltration by histiocytes with ALK expression by immunohistochemistry (1). Ten years later, the same group added seven more cases which included patients with a wider age range and broader clinical manifestations (2). A few additional cases have been reported that included localized and systemic involvement (3–5). In general, systemic disease is more common in infants and neonates (2,3). The classic presentation includes anemia, hepatosplenomegaly, and thrombocytopenia (2). Tissue biopsy shows a variable degree of histiocytic infiltration in the involved organs. The bone marrow usually shows subtle involvement although overt infiltration can be seen (2,3). Systemic involvement of other organs has been reported, including the kidneys, lungs, bone and intestine (2,3) where symptoms reflect the organ involved. Despite the apparently complicated multi-organ clinical manifestations, gradual spontaneous recovery usually occurs, sometimes with a possible role for chemotherapy (2). Death from the disease can rarely happen (2). On the other hand, localized disease usually affects older children and adults with an age range of 2-50 years. The reported sites include the skin (2), breast (2), soft tissue of the foot (2), central nervous system (2,4) and appendix (5). Generally, localized disease shows no evidence of local recurrence after surgical resection, when it is feasible (2,4).

The histiocytic infiltrate in the reported cases shared almost consistent histopathological and cytological features. For most cases, the histiocytes show abundant eosinophilic, glassy cytoplasm that may show fine vacuoles. Occasionally, histiocytes can show grayish foamy-to-fluffy cytoplasm (3) and can be multinucleated (2). Typically, the nuclei are clefted or lobulated and show irregular folding in the nuclear membrane with fine chromatin and small nucleoli (2). Although the nuclear features are usually typical, rare cases can have oval to round nuclei without foldings or grooves (2). Occasionally, cells may show emperipolesis, phagocytosis of red blood cells or polymorphonuclear leukocytes (2). Touton-type multinucleated giant cells can be seen. Some variation in the pattern of histiocytic infiltration has been reported in the form of spindling of the histiocytes with associated fibrosis and a storiform pattern (2,3). When the liver is involved, the cells are present predominantly in the liver sinusoids with variable portal tract involvement (1–3). The bone marrow can show focal, patchy or diffuse involvement (1–3).

By immunohistochemistry, the histiocytes are positive for histiocytic markers (Factor XIII, CD68, fascin and CD163) with variable expression of S100. They are consistently negative for CD1a and BRAF V600E (1–5). The ALK-1 immunostain is uniquely positive in a cytoplasmic and/or membranous pattern (1–5). The ALK1 expression by immunohistochemistry corresponds to translocation involving the *ALK *gene, most commonly with *KIF5B* which was initially described by Chan et al. (1). This translocation can be detected with either fluorescent in situ hybridization using a split apart probe for ALK or by RT-PCR and next generation sequencing which identifies the fusion partner. Despite being the most common fusion partner, other genes have been reported beside KIF5B, including TPM3 (1,3) and COL1A2 (2). Interestingly, the reported case with *ALK-COL1A2* gene fusion showed the less common nuclear features of oval nuclei with distinct nucleoli and no irregularities or folds in the nuclear membrane (2).

In general, the constellation of clinical presentation and pattern of involvement, morphology, immunohistochemistry and molecular testing help in excluding the other main differential diagnoses (6,7). The differential diagnosis includes other histiocytic disorders including Erdheim-Chester disease, Rosai-Dorfman disease, Langerhans cell histiocytosis, and localized or disseminated juvenile xanthogranuloma. A summarized detailed description of the clinical, pathological and molecular findings of these diseases is provided in Table 1.

**Table 1:** Clinical, pathological and molecular features of ALK positive histiocytosis and other histiocytic entities.

**Disease**	**Clinical features**	**Morphology**	**IHC**	**Molecular findings**
**Erdhiem-Chester**	Middle age, skeletal, cardiovascular and less CNS and retroperitoneal involvement	Histiocytes with single small nuclei and foamy cytoplasm which maybe eosinophilic. A few Touton cells. Fibrosis is present in most cases.	CD68 (+) CD163 (+) Fascin (+) S100 (-) CD1a (-) ALK-1 (-)	BRAF(V600E) mutation in >50% of cases.
**Rosai-Dorfman**	Second decade, massive bilateral cervical lymphadenopathy, fever, leukocytosis, elevated ESR, polyclonal hypergammaglobulinemia	Dilation of the lymph sinuses. Numerous cells of histiocytic appearance with a large vesicular nuclei and abundant clear or lightly eosinophilic cytoplasm. Emperipolesis is a constant feature.	CD68 (+) S100 (+) CD1a (-) ALK (-)	No specific molecular finding.
**Disseminated juvenile xanthogranuloma**	Child, skin, soft tissue lesions, Liver lesions with typical portal infiltrate sparing the biliary system (3).	Small and oval cells with a bland round to oval nuclei without grooves, pink cytoplasm and Touton multinucleated cells.	CD68 (+) CD163 (+) Fascin (+) S100 (-) Langerin (-) CD1a (-) ALK-1 (-)	No specific molecular finding.
**Langerhans cells histiocytosis**	Child, localized or systemic disease usually involving the bone, skin, liver and spleen.	Oval cells with grooved, folded, indented, or lobed nuclei with fine chromatin, inconspicuous nucleoli, and thin nuclear membranes. Frequently accompanied by eosinophils.	CD1a (+) Langerin (+) S100 (+) CD68 (+)	BRAF (V600E) in 50% of cases.
**ALK positive histiocytosis**	Infants, children or adults. Usually systemic in infants and children. Can be localized or organ confined.	Large cells with abundant usually eosinophilic to foamy cytoplasm, commonly irregular folded and maybe notched nuclei and less commonly rounded to oval in shape with smooth contour. Emperipolesis and Touton-like multinucleated giant cells can be seen.	ALK (+) S100 (+/-) CD68 (+) Fascin (+) CD1a (-) BRAF V600E (-)	*KIF5B-ALK*, *TPM3-ALK* and *COL1A2-ALK* gene fusions.

A benign localized subglottic histiocytic lesion harboring the *KIF5B-ALK* fusion that clinically mimicked subglottic infantile hemangioma was recently reported by Wolter et al. (8). They described an infiltrate of histiocytic cells with a mildly vacuolated and a slightly eosinophilic cytoplasm with small round nuclei and no nucleoli. There was no emperipolesis nor phagocytosis and the histiocytes were mixed with spindle cells. No Touton-type multinucleated giant cells were seen. The histiocytes but not the spindle cells were positive for ALK1 immunostain in an interesting pattern not described before: a perinuclear or cytoplasmic dot-like pattern. Those features, despite being partially present in some of the reported cases (2,3), prompted them to call the condition juvenile xanthogranuloma variant, ALK positive with *KIF5B-ALK* gene fusion. Whether this condition truly represents a variant of juvenile xanthogranuloma or a continuation of the spectrum of ALK-positive histiocytosis is debatable, although we favor the latter.

To the best of our knowledge, our case is the first to involve the sensory nerve root in the filum terminale of an adult patient, thus expanding the clinical manifestation of this disease. It also shows a spectrum of nuclear features that ranged from the classically folded nuclei to the less common rounded and smooth contoured. Central nervous system involvement has been described as a localized disease involving the cavernous sinus (2), the cerebellar vermis and cerebral cortex (4), and as part of a systemic involvement (9). All localized cases were in children (15, 7 and 10 year old, respectively) whereas the systemic case reported a 40-year-old male who presented with hepatic parenchymal involvement, peritoneal and omental lesions, osseous lesions, and scattered subcutaneous nodules along with a homogenous lesion in the cervical cord at the level C3 to C4 (9). The reported symptoms for the localized cases included headache, vomiting and refractory seizures (4). Our patient presented mainly with progressive lower limb weakness. The reported outcome for localized disease is good with no local recurrence after surgical resection. However, some cases such as the cavernous sinus case may not be amenable to surgical resection (2). Interestingly, this patient showed excellent response to ALK-inhibitor therapy (crizotinib) with no disease after 6 months (2). Response to ALK-1 inhibitors has also been reported in *KIF5B-ALK1* histiocytosis identified in two adults with liver and skin involvement (10) and with the case who had CNS involvement with systemic disease (9).

In conclusion, we presented the case of a 27-year-old male who had ALK-positive histiocytosis involving the sensory nerve root in the filum terminale. This is the first reported localized case occurring in the nerve root of an adult patient, thus expanding the clinical manifestation of this newly described entity. It also shows a spectrum of nuclear features in the same case ranging from the folded to the smooth-rounded nuclei. ALK-positive histiocytosis is characterized by expression of ALK1 by immunohistochemical stain and shows fusion of the *ALK* gene with different partners, most commonly *KIF5B*. An integrated histological, immunohistochemical and molecular approach is recommended during the workup of histiocytic proliferative disorders. Of particular importance is ALK1, which we propose to be performed on all cases, not only to detect this probably under-recognized entity but also because of the therapeutic benefit that may be obtained from the use of ALK inhibitor therapy especially in unresectable or systemic diseases.

## Conflict of INTEREST

The authors declare no conflict of interest.
